# Population distributions of single-cell adhesion parameters during the cell cycle from high-throughput robotic fluidic force microscopy

**DOI:** 10.1038/s41598-022-11770-z

**Published:** 2022-05-11

**Authors:** Ágoston G. Nagy, Nicolett Kanyó, Alexandra Vörös, Inna Székács, Attila Bonyár, Robert Horvath

**Affiliations:** 1 Nanobiosensorics Laboratory, Institute of Technical Physics and Materials Science, Centre for Energy Research, Budapest, Hungary; 2 Department of Electronics Technology, Faculty of Electrical Engineering and Informatics, Budapest University of Technology and Economics, Budapest, Hungary

**Keywords:** Biophysics, Motility, Nanoscale biophysics, Soft materials, Biomaterials, Biomaterials - cells, Cancer screening

## Abstract

Single-cell adhesion plays an essential role in biological and biomedical sciences, but its precise measurement for a large number of cells is still a challenging task. At present, typical force measuring techniques usually offer low throughput, a few cells per day, and therefore are unable to uncover phenomena emerging at the population level. In this work, robotic fluidic force microscopy (FluidFM) was utilized to measure the adhesion parameters of cells in a high-throughput manner to study their population distributions in-depth. The investigated cell type was the genetically engineered HeLa Fucci construct with cell cycle-dependent expression of fluorescent proteins. This feature, combined with the high-throughput measurement made it possible for the first time to characterize the single-cell adhesion distributions at various stages of the cell cycle. It was found that parameters such as single-cell adhesion force and energy follow a lognormal population distribution. Therefore, conclusions based on adhesion data of a low number of cells or treating the population as normally distributed can be misleading. Moreover, we found that the cell area was significantly the smallest, and the area normalized maximal adhesion force was significantly the largest for the colorless cells (the mitotic (M) and early G1 phases). Notably, the parameter characterizing the elongation of the cells until the maximum level of force between the cell and its substratum was also dependent on the cell cycle, which quantity was the smallest for the colorless cells. A novel parameter, named the spring coefficient of the cell, was introduced as the fraction of maximal adhesion force and maximal cell elongation during the mechanical detachment, which was found to be significantly the largest for the colorless cells. Cells in the M phase adhere in atypical way, with so-called reticular adhesions, which are different from canonical focal adhesions. We first revealed that reticular adhesion can exert a higher force per unit area than canonical focal adhesions, and cells in this phase are significantly stiffer. The possible biological consequences of these findings were also discussed, together with the practical relevance of the observed population-level adhesion phenomena.

## Introduction

Cellular adhesion plays a major role in development, differentiation, motility, migration, survival, and cancer propagation^1,2^. The protein compositions and quantities responsible for the cellular connections are determined by cell types and the environment influencing the genetic expression of cells, and they can be divided into cell–cell and cell–matrix junctions^3^. Cell–cell junctions include tight-junctions, adherens junctions, desmosomes, and gap junctions, while cell–matrix connections are hemidesmosomes and focal adhesions. The desmosomes and hemidesmosomes are connected to the intermediate filaments, and the adherens junctions and the focal adhesion are in connection with the actin filament network of the cell^4^.

Recently, a novel class of adhesion was discovered, the so-called reticular adhesions. These are characteristics of cells in the mitotic (M) phase during the cell cycle. Importantly, reticular adhesion complexes differ from canonical focal adhesion by having no vinculin inside the adhesion complexes, a protein binding the actin filament network to the integrin molecules in focal adhesion complexes. The sizes of reticular adhesion complexes are larger compared to focal adhesion complexes^5^. Computer-controlled micropipette (CCMP) and label-free single-cell biosensor measurements revealed significantly less overall adhesivity for cells adhering in the M phase^6^. Moreover, the biosensor data suggested that cells adhering with reticular adhesions have a substantially less cell mass per unit area close to the substratum than that of cells adhering in other cell-cycle phases^6^. However, the exact force values or the adhesion force per unit cell contact area of reticular adhesion still remained an interesting open question.

Single-cell adhesion measurement elucidates the mechanism and purposes of focal adhesions. The investigation of the cellular adhesion processes has particular importance in determining the validity of adhesion promoting substrates, cell vitality, and cancer cell behavior in normal conditions and under the influence of novel drug candidates. An excellent method to precisely measure the adhesion strength is the single-cell force-spectroscopy (SCFS), which was initially developed on atomic-force microscope (AFM) platforms^7–9^. These experiments, however, had several limitations, including low measurement numbers (a few cells per day) and the difficult and time-consuming cell attachement/detachment methodology. The detachment process required to attach cells to the utilized silicon–nitride cantilever with chemical bonding and then push the cells with the AFM instrument to the substrate, just to rip up the cells a few seconds later from the bottom^7,10^. Clearly, this approach has severe limitations when studying mature adhesion contacts requiring more time to form. Since these limitations heavily influenced the outcome of the experiments, a novel approach was proposed, resulting in the development of the fluidic force microscope (FluidFM)^11^. However, AFM-based force-spectroscopy investigations revealed plenty of important information about the rupture of covalent bonds and receptor-ligand interactions and unfolding of proteins^12^, which form the strong basis of current and future SCFS investigations. The traditional FluidFM technique, mounted on conventional AFM instruments, applies hollow silicon–nitride cantilevers connected to a fluid reservoir and controlled by a pressure control system, which allows the disposal or suction of fluids in a femtoliter scale^11,13,14^. FluidFM and its precise fluid manipulation technique made it possible to perform SCFS measurement on cells or bacteria firmly attached to the substrate^15–24^, colloidal force spectroscopy^19,25,26^, and 2D-3D microprinting^27,28^, with exciting techniques emerging rapidly in the field^29,30^. The latest advancement in the field of FluidFM technology is the robotized version of the FluidFM instrument (robotic FluidFM), which has a motorized, large area, and partially automatic controlled XY-stage, on which the sample is observable with an optical microscope, enabling high-throughput SCFS recordings^15,16^. This novel development tackled all previous issues with AFM-based SCFS recordings, and it became possible to measure a large population of cells in a high-throughput manner. SCFS recordings produce the characteristic force-distance curves (FD-curves) that plot the measured force values exerted by the cell and its substrate in the function of the cantilever's movement with respect to the substrate. Therefore, the robotic FluidFM technology is a robust and ideal instrument to study substrate-cell interactions, cellular development, and the behavior of cancer cells on various surfaces^15–19,31^. Robotic FluidFM was also successfully employed to calibrate high-throughput single-cell optical biosensors in order to first measure the real-time adhesion force kinetics of large cell populations^15^. The technology also opened up the possibility of high-throughput colloidal force spectroscopy measurements, and to calibrate the recorded vacuum values of CCMP to adhesion force^26^.

An important question in the field of mechanobiology is how the cell cycle influences the adhesion of cells. The phases of cell mitosis are regulated by evolutionary conserved genes and proteins, whose concentration is elevated or decreased at a specific stage of the cell cycle. To investigate cell cycle-related behavior, the cell's life cycle phase must be accessed. The cell phases follow one another, and their change requires the expression and translation or degradation of phase-related proteins. Interphase has three major phases: during the G1 phase, the cell grows and produces cellular components responsible for initiating DNA and chromosome duplication; in the S phase, the DNA is replicated; and the G2 phase produces cellular components sufficient for the daughter cells^32^. After these phases, the process of mitosis (M phase) divides the cell and establishes two identical daughter cells that can either go forward and commence the cell cycle again, starting with the G1 phase, or enter cell cycle arrest known as G0. The interphase preceding the mitosis is necessary to establish and keep up healthy cell function, for which the checkpoint kinase proteins are mainly responsible at the end of each phase^33,34^. In cancer, these proteins may malfunction, and the cell proceeds in the cell cycle despite having incompleted or multiplied chromosome replications, which leads to enhanced mutation capability^33^. To study the cell cycle, a possibility is to use the chemical arrest of the specific phases or fixate living cells and use antibodies for immunocytochemical investigation. However, there is also an option to use the fluorescent ubiquitination-based cell-cycle indicator (Fucci) construct, which is used to determine a live cell's life stage by expressing fluorescent proteins during the different phases of the cell cycle^35^. The widely used cancerous HeLa cell line was genetically engineered to express the Fucci construct, where red fluorescent protein mCherry is fused to Cdt1 expressed in the G1 stage, and green fluorescent protein Azami Green is fused to Geminin expressed in the S/G2/M stages of the cell cycle. Cdt1 and Geminin are degraded after the G1 and M phase by ubiquitination, respectively, enabling the continuous monitoring of cell cycle phases and the processes regulated by them^35^. This unique characteristic of the HeLa Fucci cell line enables the continuous monitoring of the cell cycle without labeling cells with additional chemicals or proteins.

As mentioned, previous SCFS techniques did not produce a sufficient data output, and even low-cell-number SCFS studies suggested that the measured SCFS parameters do not follow a specific distribution. Namely, the maximal single-cell adhesion force (*F*_max_), adhesion energy (*E*_max_), and the traveled distance of the cantilever, with respect to the surface at *F*_max_ (*D*_max_) were typically investigated^7,10,19,21,36^. Importantly, low throughput does not allow studying the distributions of large cell populations or revealing possible subpopulations.

Here, we first demonstrate that the employed robotic fluidic force microscopy offers an excellent platform for in-depth investigation of these single-cell mechanical parameters at the population level. We found that the main mechanical parameters of single-cell adhesion and the single-cell area (*A*_cell_) follow lognormal distributions in the various stages of the cell cycle. These parameters and their distributions have been investigated, and their differences in different cell cycle stages were determined. Cell cycle dependent adhesion was measured previously using AFM^10^, traction force microscopy^37,38^, computer-controlled micropipette, and single-cell resonant waveguide grating (RWG) biosensors^6^ to which findings our results were compared in the discussion, with special focus on the unique reticular adhesion in the M phase.

## Results

**Figure 1:**
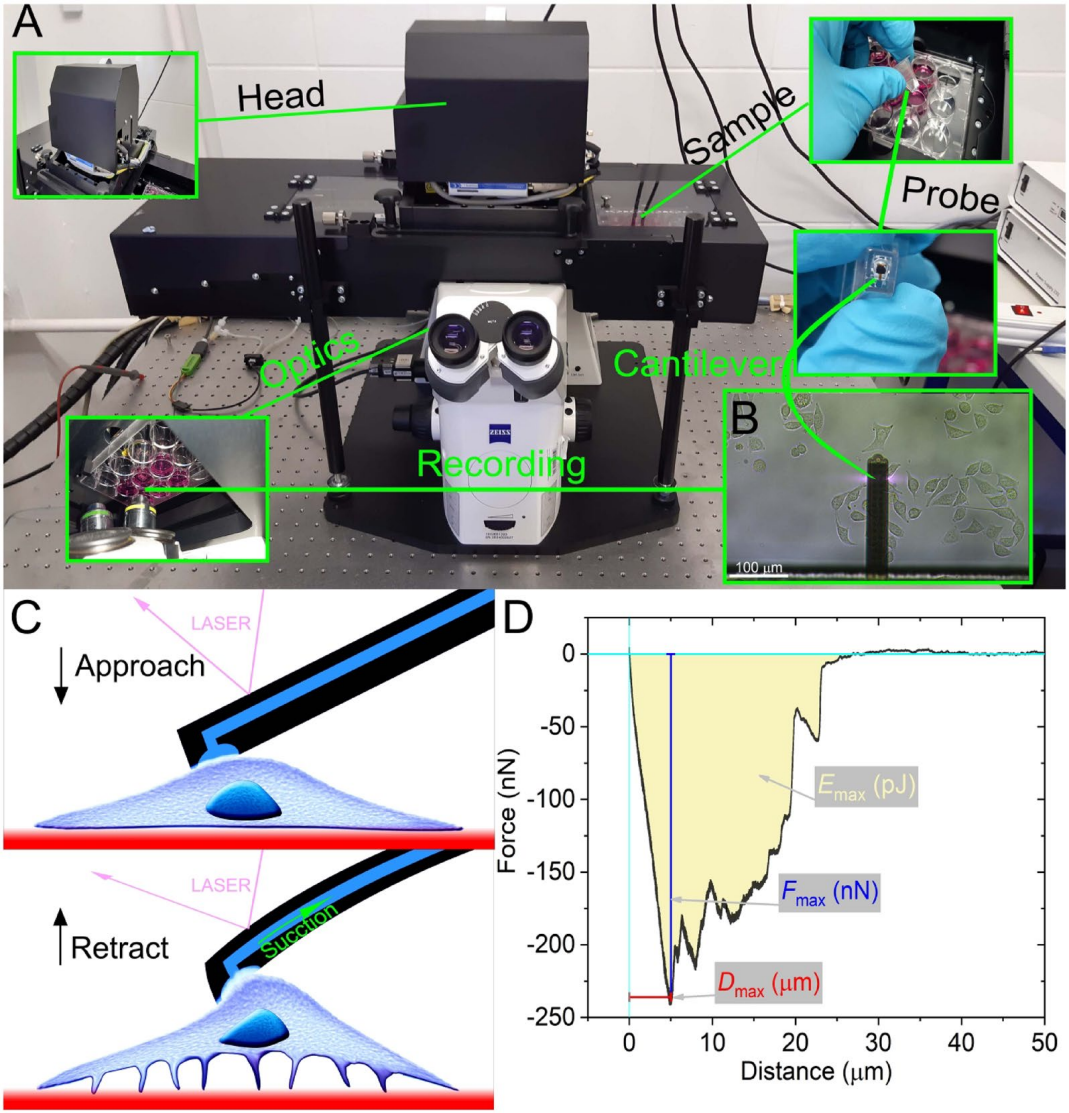
Schematic representation of the measurement setup and procedure. On the FluidFM (**A**), living cell cultures can be observed with an optical microscope **(**see insert** B** where the cantilever is clearly visible**)**. The large area sample stage under the measurement head allows multiple cell targeting in cm scale areas. During SCFS recording, cells are approached with the hollow FluidFM cantilever, which pauses upon contact with the targeted cell, and suction (vacuum) is applied to attach the cell to the aperture, after which the cantilever is retracted from the substrate (**C**). The SCFS measurement process yields the characteristic FD curves, presenting the primary parameters *F*_max_, *E*_max,_ and *D*_max_ (**D**).

**Figure 2:**
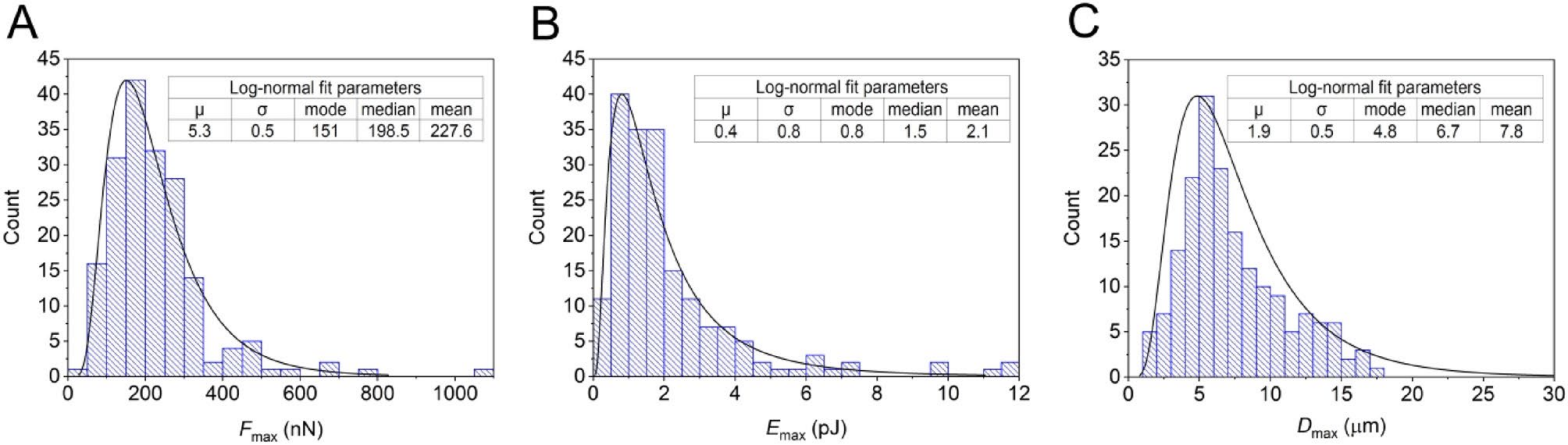
Force-distance curve parameters follow lognormal distribution for the overall population of 181 cells. Characteristic parameters recorded by SCFS are *F*_max_ (**A**), *E*_max_ (**B**), and *D*_max_ (**C**), which provide information on the adherence of single cells. The lognormal fit parameters are summarized in the tables next to the histograms of individual parameters.

Robotic fluidic force microscopy enables the detachment of single cells strongly adhered to a substrate in a high-throughput manner. Figure 1 summarizes the employed robotic FluidFM measurement procedure and the actual measurement setup. The hollow cantilever together with the targeted cells is also shown in Fig. 1C. A typical force-distance curve recorded together with the adhesion parameters provided by the instrument is highlighted in Fig. 1D. During the single-cell experiments, 251 cell adhesion curves were recorded, from which 26 cells were discarded due to weak or plain adhesion curves. It is important to note that 44 cells from 251 recordings were excluded from the final investigations since they presented multiple nuclei. This feature is outside of the main scope of our work since the role of multiple nuclei in cell adhesion is not fully understood yet. Also, the low number of polynuclear cells measured did not allow the proper investigation of population distributions and significant differences between populations when the cells with different colors were separated. However, the corresponding results are presented in the Supplementary Information (SI) (see Figs S5–7) file when multi-nuclei cells are treated as a sub-population. Of note, including multi-nuclei cells in the single-nuclei population did not influence the obtained significance levels and our main conclusion. (But they have been discarded regardless of the total number of cells in-depth analyzed, resulting in a total number of 181 investigated cells.) The distributions of the adhesion parameters from 181 cells, regardless of their color, are shown in Fig. 2. Typical parameters of FD-curves were evaluated for the general population, and lognormal distribution was the adequate fit for these characteristic parameters. The lognormal nature of the obtained data is an important finding since most prior literature, with a relatively low number of single-cell adhesion measurements, treats experimental data as normally distributed or without the intention of addressing the nature of adhesion parameter distribution^7,19,36,39^. Since in a lognormal distribution a significant portion of the cells has parameters far from the mode, conclusions based on adhesion data of a low number of cells (or treating the population as normally distributed) can be heavily misleading.

We found that HeLa Fucci cells yielded adhesion forces (*F*_max_) in the range of 25–1065 nN, adhesion energies (*E*_max_) in the range of 0.06–11.9 pJ, and detachment distances (*D*_max_) in the range of 1.2–17.5 µm. (see Fig. 2).

The final number of cells for the single-nuclei evaluation was 181, separated into 38 red, 43 yellow, 44 green, and 55 colorless cells. The measured single-cell parameters were visualized in the form of scatter plots in order to highlight the measured range of various adhesion parameters and to reveal possible correlations. The correlation of basic SCFS parameters is as expected for *F*_max_ *vs* *E*_max_ based on the literature^15,16^ (see SI Fig. S1 and S2).

**Figure 3:**
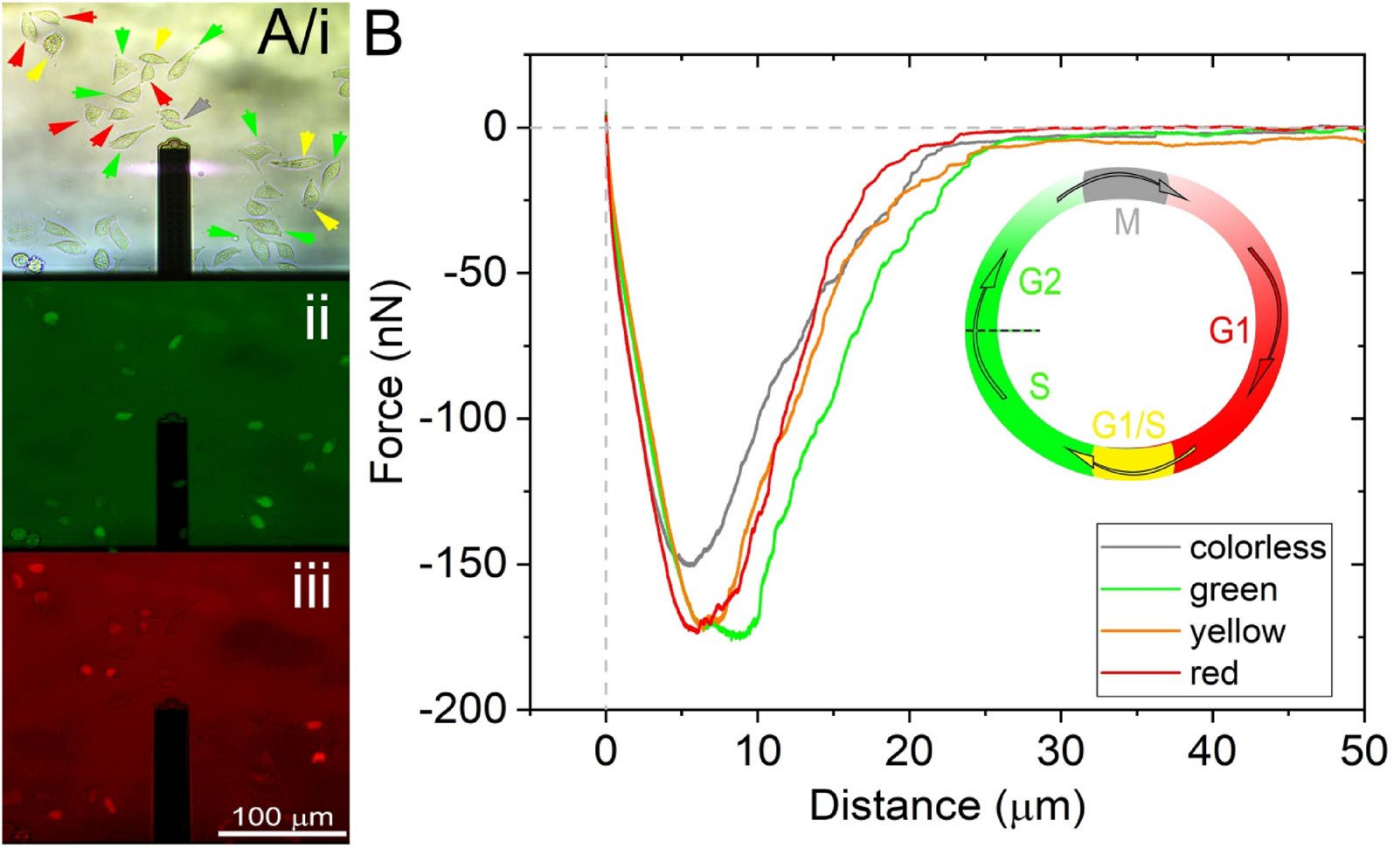
The Fucci construct and the actual measurement when the FluidFM cantilever is approaching the targeted cell. (**A**) Schematics of the HeLa Fucci cycle indicate the colors visible during the different phases. Fluorescent images were taken during SCFS measurements on the FluidFM platform. Green and red fluorescent channels were visualized simultaneously, and according to their emitted light spectra, cells were labeled as green, red, yellow (green and red), and colorless. (**B**) Averaged characteristic SCFS FD-curves were obtained from each measurement belonging to different color phases of HeLa Fucci cells. Averaged FD-curve belonging to a single set of colors were calculated based on the position dependency (distance) of the force values of individual recordings at the same position. For the averaged FD-curves, 38 red cells, 43 yellow, 44 green, and 55 colorless cells were averaged, respectively. The inset shows the cell cycle phases and the actual cell colors.

The microscope image of typical HeLa Fucci cells is shown in Fig. 3A. The recorded force-distance curves were averaged for the cells with different colors and are shown in Fig. 3B. It is already revealing that the averaged curve corresponding to the colorless cells has a different character, with clearly visible smaller *F*_max_, *E*_max,_ and *D*_max_ values. Also, averaged FD-curves of green cells show elevated SCFS parameters, while red and yellow cells FD-curves seem to be alike on average.

**Figure 4:**
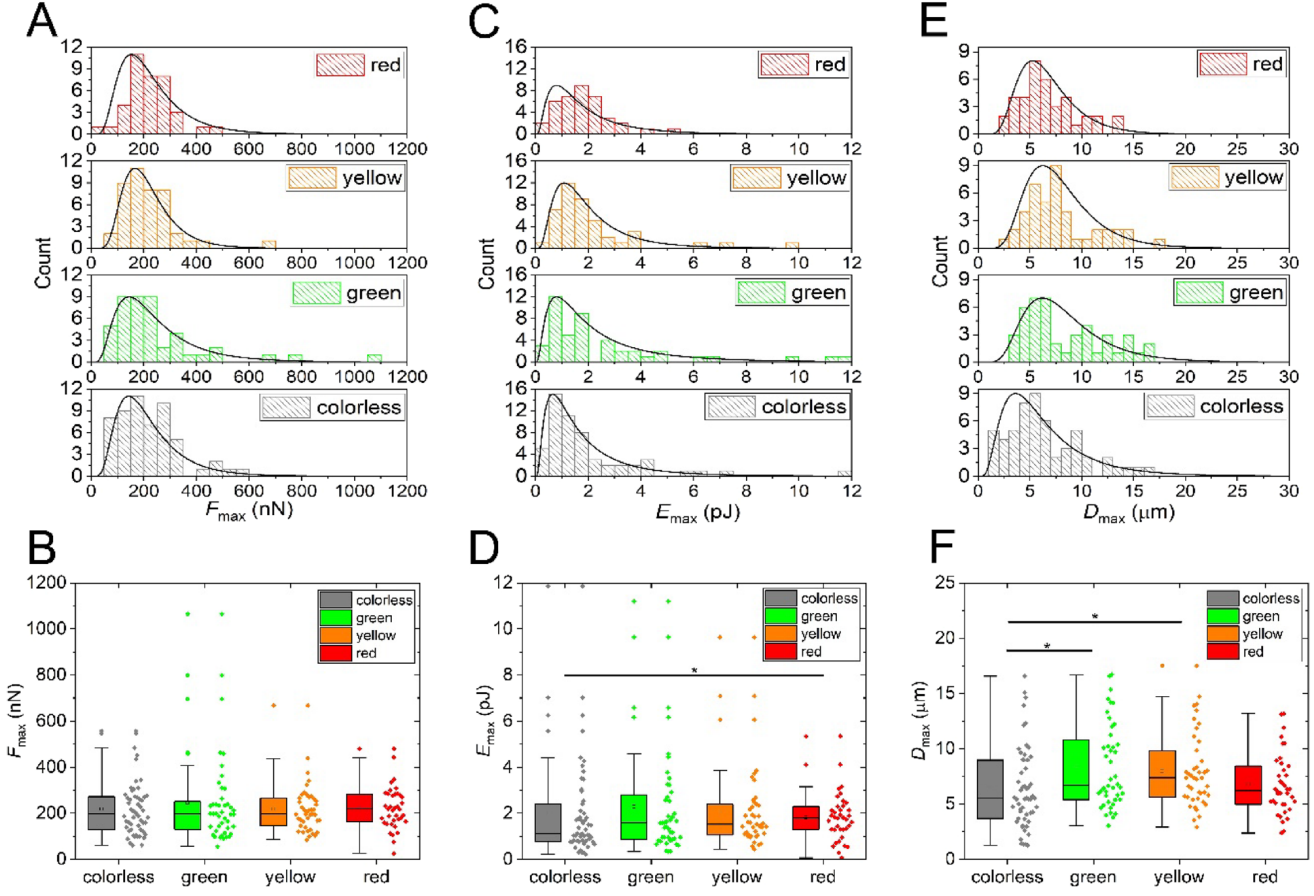
Adhesion parameter distributions with respect to cell color are presented on histograms with lognormal distribution (**A,C,E**) and box charts (**B,D,F**). Parameters *F*_max_ (**A,B**) and *E*_max_ (**C,D**) show no statistical difference between individual phase colors (except for a weak significance for the colorless-red *E*_max,_ see Table S1), but *D*_max_ (**E,F**) presents a difference between colorless (M phase), green (G2 phase), and yellow (S phase) cells (*p* < 0.05). *F*_max_: colorless cells mean = 218 ± 16 nN; green cells mean = 252 ± 29 nN; yellow cells mean = 218 ± 16 nN; red cells mean = 222 ± 15 nN. *E*_max_: colorless cells mean = 1.9 ± 0.3 pJ; green cells mean = 2.6 ± 0.4 pJ; yellow cells mean = 2.1 ± 0.3 pJ; red cells mean = 1.8 ± 0.2 pJ. *D*_max_: colorless cells mean = 6.4 ± 0.5 µm; green cells mean = 6.4 ± 1.8 µm; yellow cells mean = 8 ± 0.5 µm; red cells mean = 6.8 ± 0.5 µm.

Next, single-cell data corresponding to the different colors was analyzed separately, and the results are summarized in Fig. 4. Importantly, distribution data of subpopulations are also well fitted by lognormal distributions. In-depth analysis revealed that the *F*_max_ and *E*_max_ parameters have a cell-cycle independent character; no significant differences were found. All parameters were tested for normal and lognormal distributions. Although normal distribution was found to fit well on the red cell population, especially in the *F*_max_ and *E*_max_ parameters, the significance levels were tested with a non-parametric significance test due to the overall lognormal distribution found in the general population and a large number of single-cell measurements. However, the *D*_max_ parameter has a significantly different distribution for the colorless cells. Basic adhesion parameters *F*_max_ and *E*_max_ show no differences in the various stages of the cell cycle (expect the colorless-red weak significant difference in *E*_max,_ see Table S1), but *D*_max_ has higher values in the G2 phase (green) and S phase (yellow) compared to the M phase (colorless). The Lowest *D*_max_ values were recorded during the M phase, followed by G1 (red), S, and G2 phases.

**Figure 5:**
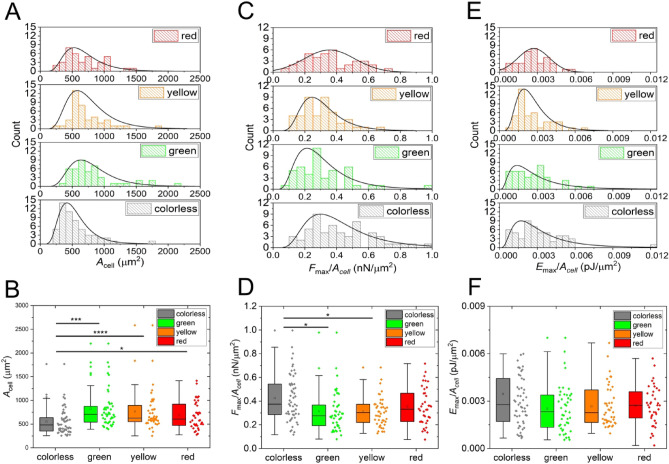
Area (*A*_cell_) and its effect on the area normalized parameters (*F*_max_/*A*_cell_, *E*_max_/*A*_cell_,) presented on histograms with fits of lognormal distributions (**A,C,E**) and box charts (**B,D,F**). A significant difference was found in the *A*_cell_ between colorless cells and all other subpopulations (**B**). Also, the *F*_max_/*A*_cell_ values between colorless cells, green and yellow cells were observed to be significantly different (**D**). *E*_max_/*A*_cell_ has not shown any difference in their distribution (**F**).

Single-cell areas were also determined as detailed in the Materials and Methods section. The distributions of the single-cell area were found to be lognormal in all cell cycle states, with the smallest mean area for the colorless cells (Fig. 5A, B). We also investigated the area normalized single-cell adhesion parameters, such as *F*_max_/*A*_cell_ and *E*_max_/*A*_cell_. Interestingly, the normalized parameter *F*_max_*/A*_cell_ is significantly higher in the M phase compared to the S phase and G2 phase, but in *E*_max_*/A*_cell,_ no evident difference is present. The corresponding distributions are also shown in Fig. 5C–F. It is important to highlight that this normalization resulted in statistically significant differences in the force data, colorless cells *F*_max_/*A*_cell_ values were largest: colorless cells mean = 0.43 ± 0.03 nN/µm^2^; green cells mean = 0.31 ± 0.03 nN/µm^2^; yellow cells mean = 0.31 ± 0.02 nN/µm^2^; red cells mean = 0.04 ± 0.03 nN/µm^2^. However, interestingly, we did not find significant differences in the population distributions of the *E*_max_/*A*_cell_ parameter. For more detailed statistical analysis, see Figure S3, 4 and Table S1, 2 with calculated p-values.

**Figure 6:**
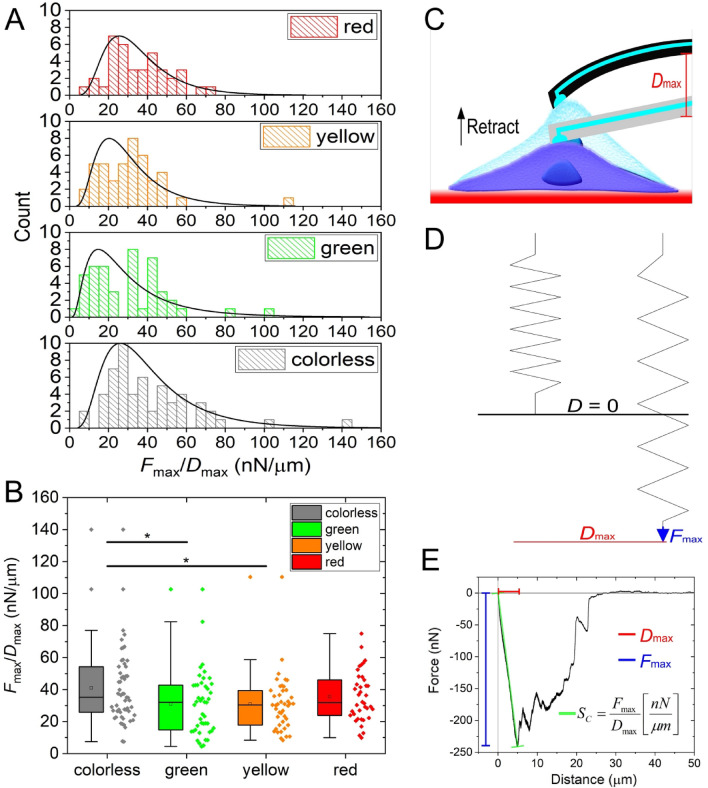
Spring coefficient (*S*_c_) characterizes the elasticity of the cell and the illustration of its physical meaning. *S*_c_ produced lognormal distributions in all color phases **(A)**, and a significant difference between colorless, green, and yellow cells **(B)**. The elastic capability of the cell shows a deformation upon pulling it from the surface with the FluidFM cantilever. Note the drawing is not in scale. In reality, the bending of the cantilever is small compared to the elongation of the cell. (**C**), which can be interpreted as the pulling of spring with a specific amount of force. The longest elongation *D*_max_ is reached when pulling the cell with the maximum force *F*_max_ (**D**). Thus the fraction *D*_max_ and *F*_max_ yields *S*_c_, which is the linear slope connecting the maximal force–elongation with the zero level (**E**).

Another promising new SCFS quantity introduced in the present work is the spring coefficient (*S*_c_), defined as *F*_max_/*D*_max_, that characterize the average elasticity of the cells. The purpose of the introduced spring coefficient is to provide a way for the convenient characterization of the average elasticity of the cells. The detachment force-curves indirectly contain this information, although they were primarily obtained to measure the adhesive properties of the cells. Of note, the cell and the cantilever can be considered as two serially coupled units and therefore, precisely, *S*_c_ by definition is the spring coefficient of the cell-cantilever system (see Fig. 6C, D). However, the spring constant of the cantilever is relatively large (2 N/m), resulting in significantly fewer cantilever deflections than cell elongations (nm vs micrometer range). Therefore, *S*_c_ with good approximation can be considered as the spring coefficient of the given cell. The resulting mean *S*_c_ values were 41.0 ± 3.2 nN/µm for colorless cells, 30.1 ± 3.0 nN/µm for green cells, 31.0 ± 2.7 nN/µm for yellow cells, and 35.6 ± 2.5 nN/µm for red cells. The corresponding distributions and significance values are shown in Fig. 6A, B.

## Discussion

The results have shown that a high-throughput FluidFM investigation of HeLa Fucci cancer cells presents a lognormal distribution in all SCFS parameters and cell area, even in the total and the sub-populations corresponding to cells with various colors. Parameters such as *F*_max_, *E*_max,_ and *D*_max_, which are characteristic of FD-curves recorded belonging to individual cells, show only significant differences in *D*_max_ between colorless and yellow or green colored cells. Since *A*_cell_ is also significantly larger in the yellow and green sub-population than in the colorless population, *D*_max_ corresponds well to how large or elastic a cell is. Importantly, in the colorless state the cells not only have a smaller contact area, but extend less due to the mechanical deformation during the detachment process. This means that during the M phase (colorless), a cells' area is reduced due to the mitosis, and reaches the *F*_max_ at shorter pulling distances than cells with a larger membrane and cytosol (e.g., yellow or green phase). Our results prove that HeLa Fucci cells also show the tendency^40^ to have higher cell areas in the S/G2 phase compared to the G1 phase, but this difference was evaluated as not significant. The main difference regarding *A*_cell_ is between the M phase (colorless cells) and all other phases, which is in tune with the literature regarding the rounding up of the cell during mitosis^6,37,38,41^.

The parameters *F*_max_, *D*_max_ and *E*_max_ are standard parameters used in single-cell force-spectroscopy literature^8^, although they are often reffered to „maximal detachment force”, „rupture distance” and „adhesion energy” ^15,19^. It is important to note, the *F*_max_ is highly dependent on the position where the cell is grabbed with the cantilever during recording, and therefore we always aimed for the nucleus region to eliminate sequential ruptures. To address the nature of smaller rupture events might be also interesting. For this purpose, the present setup is probaly not the optimal choice, since it can measure very high forces (up to 3500 nN), and therefore has a lower resolution and a larger retraction distance. Standard AFM based SCFS can however produce cleaner signals and show individual ruptures, even at the molecular scale^8^, but AFM-based methodologies (also the present FluidFM add-on systems) only provide low retraction distances (up to 10–15 µm).

Interestingly, we have found no significant statistical difference between the *F*_max_ and *E*_max_ distributions, which are investigated further by normalizing these parameters with the individual cell's area. The significant difference in the *F*_max_*/A*_cell_ parameter between colorless and green/yellow cells is a newly discovered phenomenon, which we explain with the presence and expression of αVβ5 integrins and reticular adhesion proteins during the M phase of the cell^42^. In our experiments, there was no difference in the distribution of *F*_max_ parameter among the different phases, but the mean value of *A*_*cell*_ was significantly smaller in the M phase. These findings are to some extent contrary to assumptions that cells not only round-up during mitosis, but cytokinesis requires the weakening of cell-substrate adherence^43–45^. The recently discovered reticular adhesion complexes are directed by αVβ5 integrins to enable cytoskeletal rearrangements during mitotic round-up and cytokinesis^5^. We conclude that reticular adhesion complexes exert the same amount or even more force per unit area than focal adhesions, which result highlights the important function of reticular bonds responsible for the anchoring of cells during round-up and division. The HeLa Fucci cell line was studied by a high lateral resolution single-cell resonant waveguide grating (RWG) biosensor, allowing the direct measurement of the optical density of the cell-substratum contact zone in different color phases. The previously measured lower cell mass per unit area inside the cell-substratum contact zone^6^ together with our new finding on larger force per unit area in the M phase highlight that not only the adhesion complexes but the whole cell-substratum contact zone has a different morphology in reticular adhesions compared to canonical focal adhesions (stronger, but less dense). Controversially, traction force in the late M phase was found to be lower on the substrate^38^, but as we showed it, the overall cell binding force is similar, and the exerted force per unit area is even significantly larger in colorless cells when population distributions are considered. It must be also emphasized that our above results are perfectly in tune with the morphology of reticular adhesions^5^ and highlight the differences at the population distribution level, too. Also, it must be noted that *D*_max_ recorded with the fluidic force microscope is a unique parameter not available by other methods. While the cell area has a steady growth during the cell cycle, also observed in the present work, the exerted force onto the surface was found to be similar and even to some extent larger in the green-colored (S/G_2_ phase) population. Interestingly, previously reported biphasic behavior of the cell cycle^37,46^ was undetected by us in the acquired parameters provided by SCFS. The loss in cell adhesion area in the G2 phase, as reported by Jones et al. ^46^, does not reduce the amount of force or energy exhibited by HeLa Fucci cells in our setting.

The introduced spring coefficient (*S*_c_) of a cell, derived from the fraction of *F*_max_ and *D*_max_ parameters, corresponds to the mean elasticity of a cell, and it can be visualized by adding a linear slope to the beginning of the retract phase of the FD-curves connecting the zero level to the (*D*_max_, *F*_max_) coordinates as seen in Fig. 6E. Physically, and from the view of cellular adhesion, *S*_c_ determines the overall adhesive elasticity of a cell, created by the pull on the membrane and the underlying receptor-ligand adhesive bonds, which is interpreted as the elongation of two connected springs forming a unit. Of note, by fitting a linear on the retrace curve, the obtained spring coefficient (*F*_max_/*D*_max_) provides a fair and reliable estimate on the elastic behavior of the cell—if the experimental conditions (e.g., pulling rate) are set in a way that the deformation and the cell's response can be approximated linearly. In the case of our high-throughput investigation on HeLa Fucci cancer cells, we have determined that the *S*_c_ is significantly larger in the colorless cells compared to other color phases. From the biological perspective, it means that cells in the M phase have smaller deformation capabilities, and therefore, they are stiffer. This result is in line with previous works^47–49^, however, the statistical difference with the other phases was not shown before using SCFS techniques. Our results highlight the differences in single-cell adhesion parameters in the M phase at the population level and suggest that it would be advantageous to investigate and selectively target M phase controlled protein expression to understand their role in cancer propagation better^50,51^. Drug candidates affecting adhesivity could be better and more meaningfully explored in future works based on our results.

## Materials and methods

### Cell line and relevant protocols

HeLa Fucci cells (RCB2812, RIKEN BRC) were maintained in Dulbecco's Modified Eagle's Medium (DMEM, 31,885 Gibco) supplemented with 10% Fetal Bovine Serum (Biowest SAS, France), 100 U/ml penicillin, and 100 μg/ml streptomycin mixture solution (Merck, Germany), in a humidified incubator at 37 °C and 5% CO_2_. Before the experiment cells were detached from a tissue culture Petri dish using standard protocol (0.05% (w/v) trypsin, 0.02% (w/v) EDTA solution). Cells were picked up in a 1 ml culture medium, and 50 µl of cell suspension containing ~ 1.5 × 10^5^ cells was transferred to each well of a 6-well tissue culture plate filled with a completed culture medium. The 6-well culture dish was placed into an incubator for 24 h to let HeLa Fucci cells adhere to the bottom of the dish. During measurements, only single cells were considered without contribution to large and dense clusters.

### Fluidic force microscopy measurements and related calibration

SCFS measurements were carried out using the robotized FluidFM instrument (Cytosurge AG., Zürich, Switzerland) set on a vibration-free table. *(*Note: the robotized FluidFM was called BOT^15,16,26,27,52,53^. Currently, the company titled it OMNIUM.) The 6-well plate containing the cultured HeLa Fucci cells was placed into the robotized XY-stage of the instrument. Cells were recorded for up to 4 h at room temperature in completed culture media^54^. The experimental setup requires the buffer-filled micropipette FluidFM cantilever mounted on the Z-stage of the instrument. The stage of the robotized FluidFM can handle two separate plates, from which one is the experimental plate and the other one is utilized for cleaning purposes. After each SCFS measurement on living cells, the cantilever attached to the plastic holder, known together as the probe, is dipped in MQ water followed by a few seconds of 5% hypochlorite, then rinsed again 4 times with MQ water. Before each measurement cycle, the inverse optical lever sensitivity (*InvOLS*) is determined, since it is dependent on the position of the laser reflection optics of the instrument, and its accuracy influences the obtained force values, as it was previously shown by us^52^. Another essential constant for calculating the adhesion force is the spring constant (*k*) of the cantilever, which is calculated by the built-in function of the robotized FluidFM using the Sader method as calibration strategy^55^. Obtained *InvOLS* and *k* values were always defined for a certain laser-spot position and every individual cantilever to receive the most accurate results with the smallest possible error ^52^. Before SCFS recording, the colors of the cells were determined by the fluorescent mode of the FluidFM's Zeiss microscope. Images were taken of every individual cell before the measurement to validate their emitted fluorescent color. For SCFS recording, a FluidFM micropipette cantilever with an 8 µm aperture was used, and recording was done with the following parameters: set-point 20 mV, approach and retract speed 1 µm/s, pressure − 500 mBar, pause 5 s, retraction distance 150 µm. The pulling rate was optimized for our experiments in prior. We have found the 1 um/s optimal for our goals, so this rate is fixed in our protocol (and it is also quite commonly used in the literature^15,16,56^). All of the cells and grabbing positions were selected manually before recording their adhesivity. The selection criteria were mainly that these cells should be free of any cellular contact, to measure their focal adhesions only. Moreover, all of the cells were targeted above the nucleus to inhibit sequential unbinding of receptor-ligand interactions. Note, some multinuclei cells were measured because of curiosity, but most of them were evaluated afterwards as multinuclei when carefully observing their microscopic images (See SI file for more details).

### Data evaluation

Analysis of the obtained SCFS data was carried out using a custom Matlab code written by us to evaluate the characteristic FD-curves and save all numeric parameters automatically for quicker evaluation. Cell area (*A*_*cell*_) was calculated in CellProfiler ^57^, which switches between images and saves the results automatically after the user manually draws the outline shape of the cell. Lognormal and normal distribution curve fitting, data plots, and significance tests were made in Origin 9.5. For the raw data, which was not normally distributed, the non-parametric two-sample Kolmogorov–Smirnov test was used (*α* = 0.05), and after taking the natural logarithm of the raw data two-sample t-test (unpaired, unequal variances) was applied (for results, see also the SI file, Figure S3, 4, and Table S1, 2).

## Supplementary Information

Supplementary Information.
