# Supplementary material for: Population distributions of single-cell adhesion parameters during the cell cycle from high-throughput robotic fluidic force microscopy
Source: Sci Rep. 2022 May 11;12:7747. doi: 10.1038/s41598-022-11770-z (PMC9095720; doi:10.1038/s41598-022-11770-z)
Supplement: Supplementary file 1 — Supplementary Information. [file 41598_2022_11770_MOESM1_ESM.docx]

**Supplemental Information**

**to**

**Population distributions of single-cell adhesion parameters during the cell cycle from high-throughput robotic fluidic force microscopy**

Ágoston G. Nagy^1,2^, Nicolett Kanyó^1^, Alexandra Vörös^1^, Inna Székács^1^, Attila Bonyár^2^, Robert Horvath^1^

*1) Nanobiosensorics Laboratory, Institute of Technical Physics and Materials Science, Centre for Energy Research, Budapest, Hungary.*

*2) Department of Electronics Technology, Faculty of Electrical Engineering and Informatics, Budapest University of Technology and Economics, Budapest, Hungary*

# **Adhesion parameter ranges and relations of the HeLa Fucci cell population**

SCFS data shows relations in parameters such as *F*_max_*-E*_max,_ as it was previously described in the literature. Our results for the general population of HeLa Fucci cells are shown in **Fig. S1**. The correlation between these two parameters explains the outcome for cells picked randomly from a population, where these cells are in various cell-cycle states presented here in **Fig. S2**. The relationship of *F*_max_*-D*_max_ cannot be explained with linear correlation fitting since *D*_max_ is a unique parameter describing the elastic elongation of a cell until it exerts the maximal force onto the substrate. Also, in our investigation, *D*_max_ depends on cell cycle state, which significant difference is not visible on the *F*_max_*-D*_max_ scatter plot representing the cell colors in **Fig. S2B**.


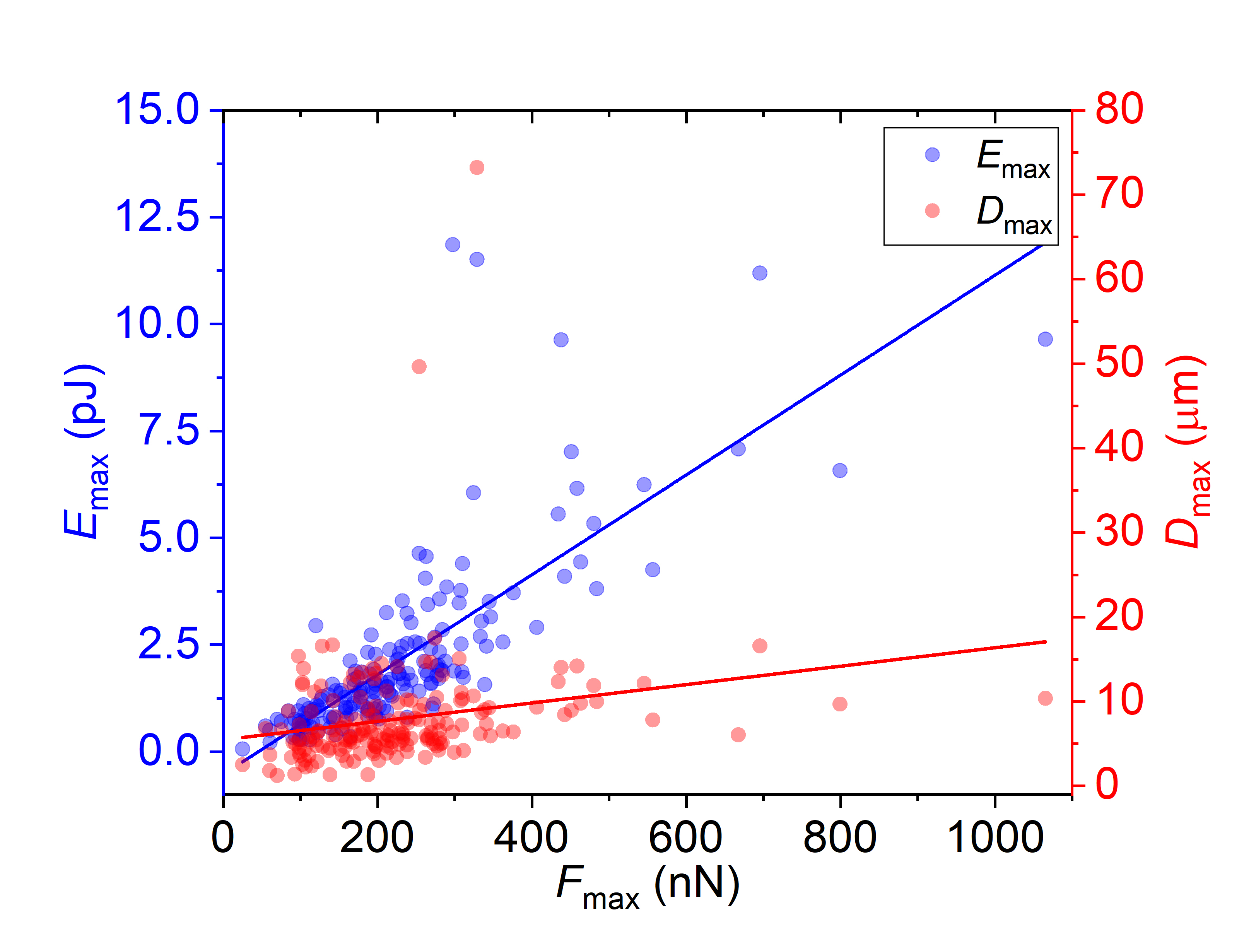


**Figure S1.** High-throughput SCFS investigation on HeLa Fucci cells revealed a correlation (R) of 0.78 between the characteristic *F*_max_-*E*_max_ parameters (blue). *F*_max_-*D*_max_ (red) correlation resulted in 0.23.


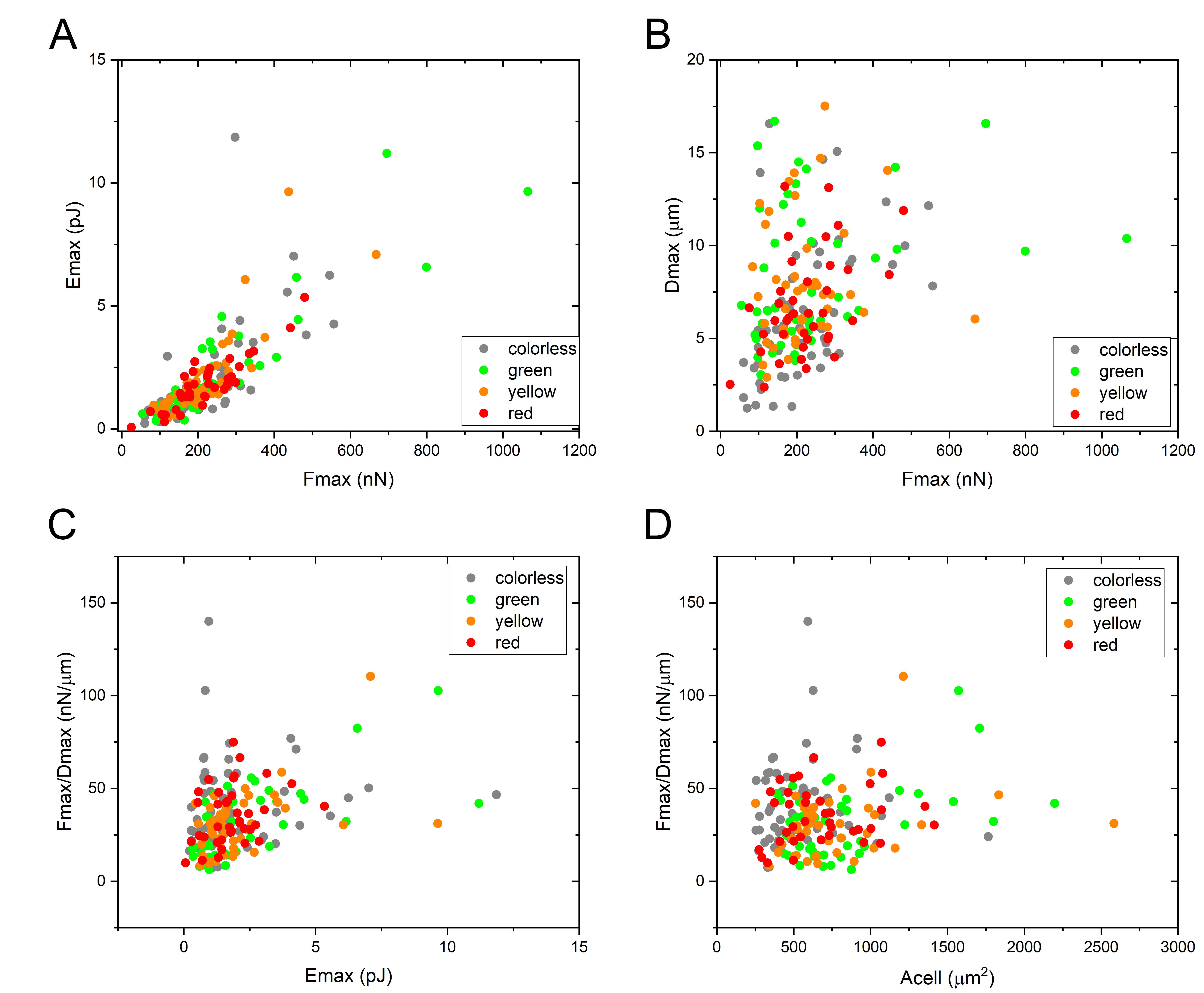


**Figure S2.** Color separated comparison of SCFS parameters and *A*_cell_. A) *F*_max_*-E*_max_, B) *F*_max_*-D*_max_, C) E_max_*-F*_max_*/D*_max_, D) *A_cell_*/*F*_max_*/D*_max_.

# **Histograms and column graphs with indicated significant differences in normalized values**

All parameters were normalized by taking the natural logarithm of the datasets, and the Student’s t-test (unpaired, unequal variances) was conducted to retrieve significant differences. No difference was found in parameters *F*_max_ and *E*_max_ (**Fig. S3A-D**), but in the parameter *D*_max,_ the same difference was observed as in the raw data with one exception, namely, red and green cells are also significantly different shown in **Fig. S3E-F**. The area (*A_cell_*) normalized values *F*_max_/*A_cell,_* and *E*_max_/ *A*_cell_ show the same significant differences as seen on the raw data (**Fig. S4A-D**), and the newly introduced spring coefficient (*F*_max_/ *D*_max_) present the same differences between the colors.


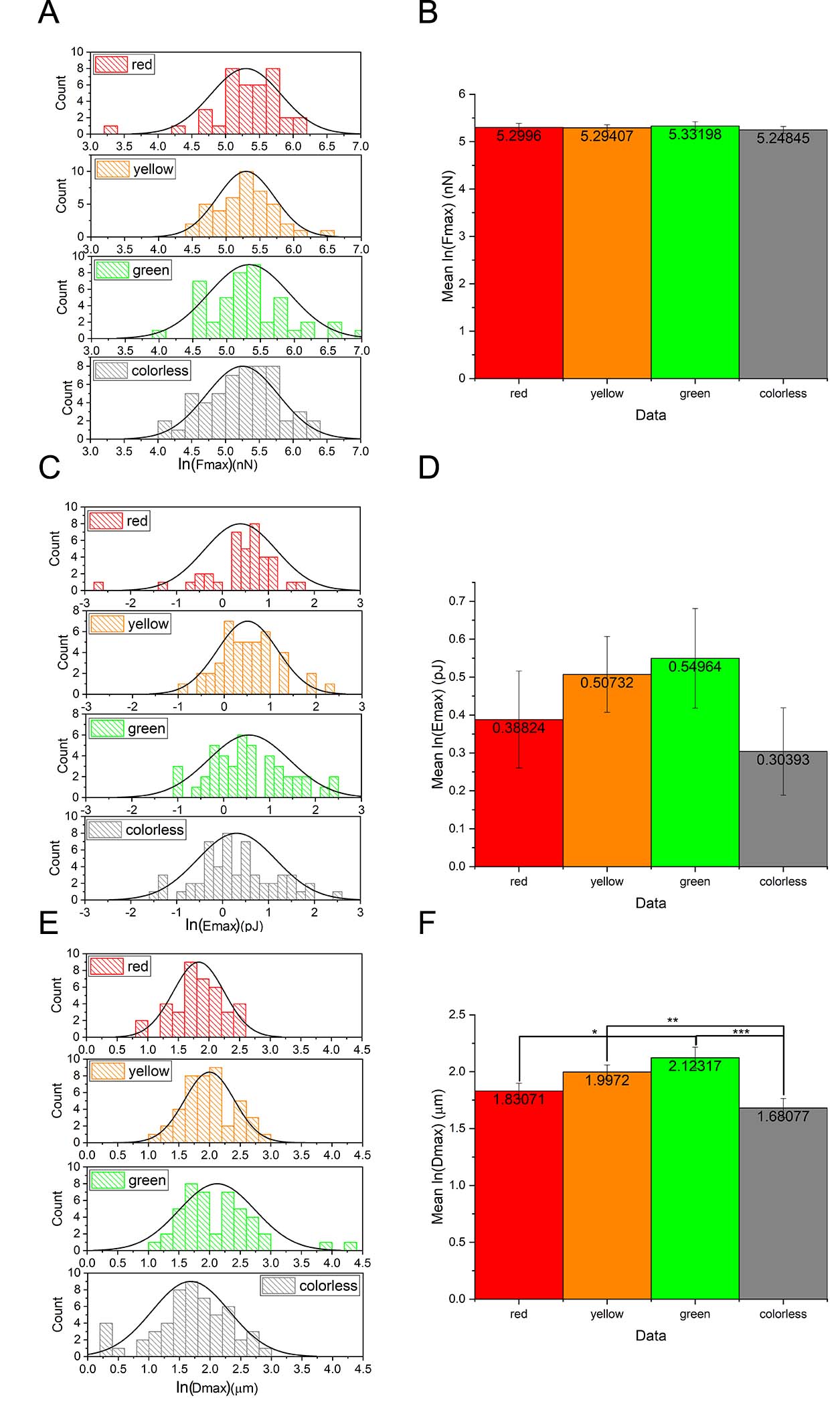


**Figure S3.** Histograms and labeled significances on parameters *F*_max_ (A,B) *E*_max_ (C,D) and *D*_max_ (E,F).


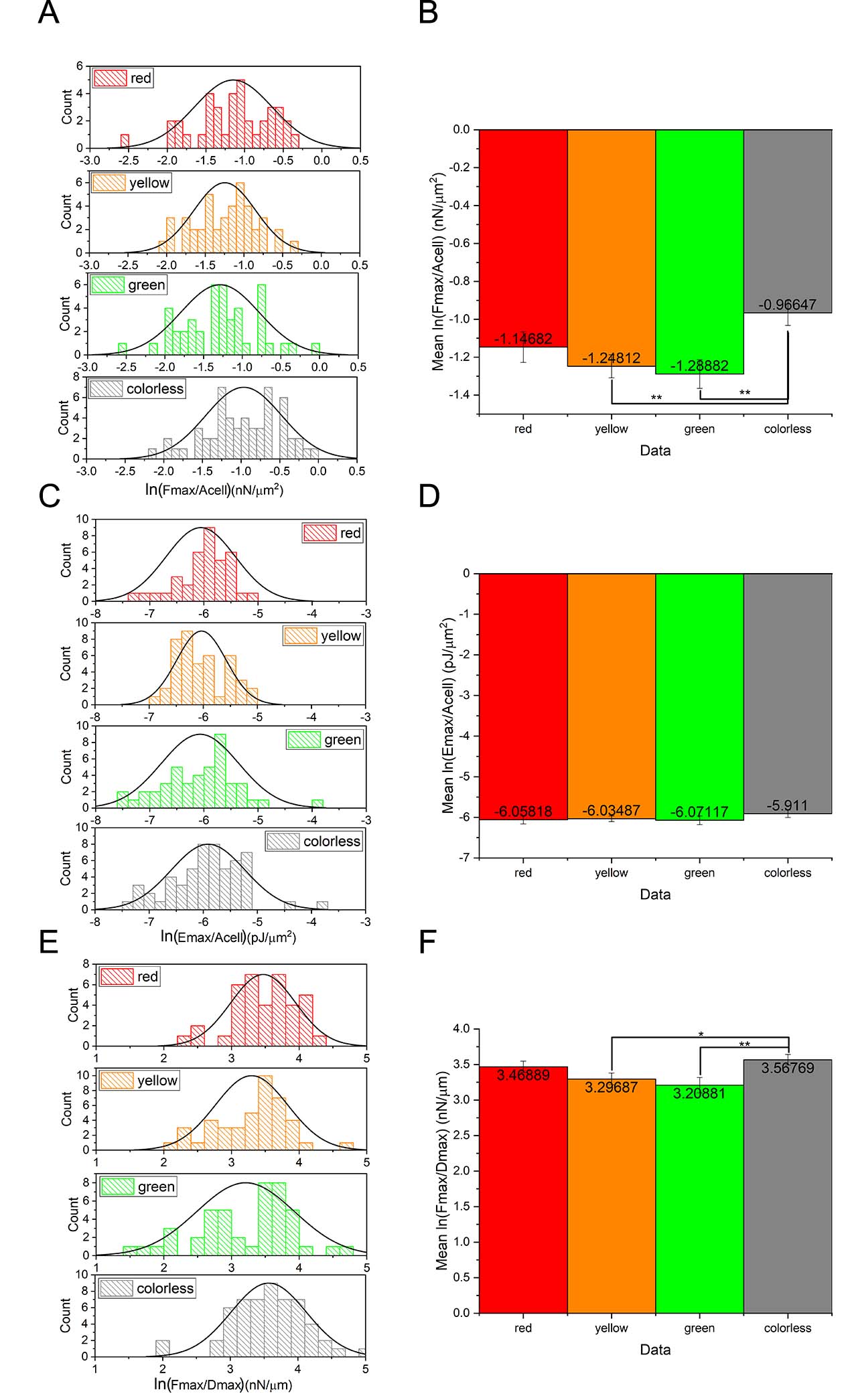


**Figure S4.** Histograms and labeled significances on parameters *F*_max_/*A_cell_* (A,B) *E*_max_/*A_cell_* (C,D) and *F*_max_*/D*_max_ (E,F).

# **Results of statistical analyzis in table format**

**Table S1.** KS-test results, p-values (* p<0.05)

|  | Fmax | Emax | Dmax | Fmax/Acell | Emax/Acell | Fmax/Dmax |
| --- | --- | --- | --- | --- | --- | --- |
| colorless-green | 0.95 | 0.38 | * 0.02 | * 0.011 | 0.51 | * 0.02 |
| colorless-yellow | 0.75 | 0.13 | * 0.013 | * 0.012 | 0.2 | * 0.02 |
| colorless-red | 0.47 | * 0.03 | 0.34 | 0.43 | 0.31 | 0.73 |
| green-yellow | 0.8 | 0.32 | 0.36 | 0.9 | 0.29 | 0.63 |
| green-red | 0.61 | 0.25 | 0.052 | 0.12 | 0.74 | 0.04 |
| yellow-red | 0.65 | 0.58 | 0.26 | 0.36 | 0.33 | 0.42 |

**Table S2.** t-test results, p-values (* p<0.05; ** p<0.01; *** p<0.001)

|  | Fmax | Emax | Dmax | Fmax/Acell | Emax/Acell | Fmax/Dmax |
| --- | --- | --- | --- | --- | --- | --- |
| colorless-green | 0.47 | 0.16 | *** 0.0006 | ** 0.0018 | 0.26 | ** 0.008 |
| colorless-yellow | 0.64 | 0.19 | ** 0.003 | ** 0.002 | 0.28 | * 0.016 |
| colorless-red | 0.65 | 0.63 | 0.17 | 0.09 | 0.29 | 0.36 |
| green-yellow | 0.73 | 0.8 | 0.26 | 0.68 | 0.77 | 0.52 |
| green-red | 0.79 | 0.38 | * 0.013 | 0.21 | 0.93 | 0.054 |
| yellow-red | 0.96 | 0.47 | 0.073 | 0.32 | 0.85 | 0.13 |

# **Histograms fitted on excluded polynuclear cells’ SCFS parameters and their area (*A*_cell_)** **normalized values**

Despite their color state, multi- nuclei cells (**Fig. S5**) have been excluded from the final evaluation since their behavior might alter the results observed in the single-core phenotype. Also, histograms created by the characteristic SCFS parameters show that the overall number of multi-nuclei cells recorded does not present a sufficient amount to investigate these cells in detail (**Figs S6-7.**).


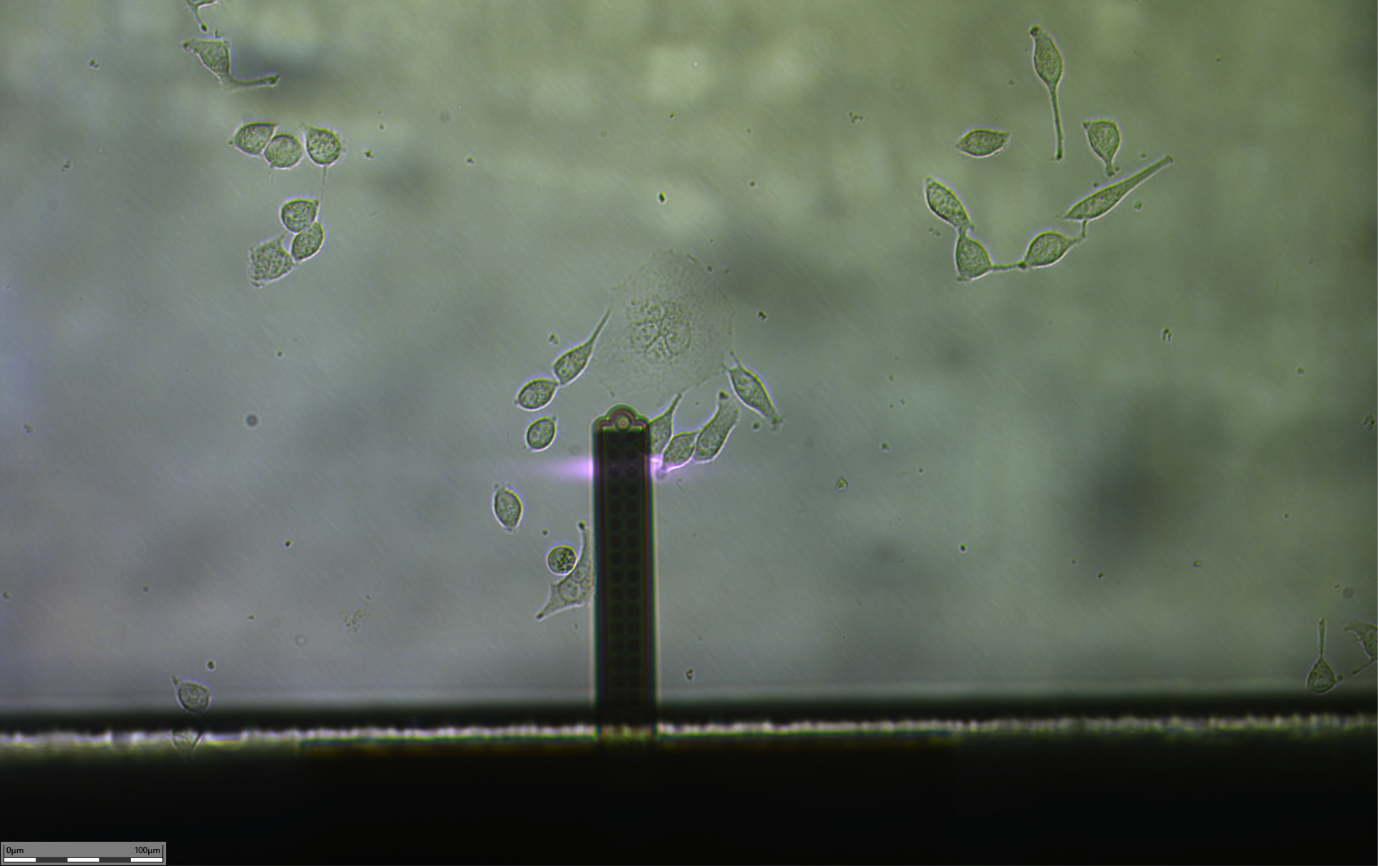


**Figure S5.** Cells with multiple nuclei (for an example see the cell at the tip of the cantilever) were excluded from the final investigations since their measurement was not the primary objective and because their role and behavior in cancer cell proliferation are unknown.


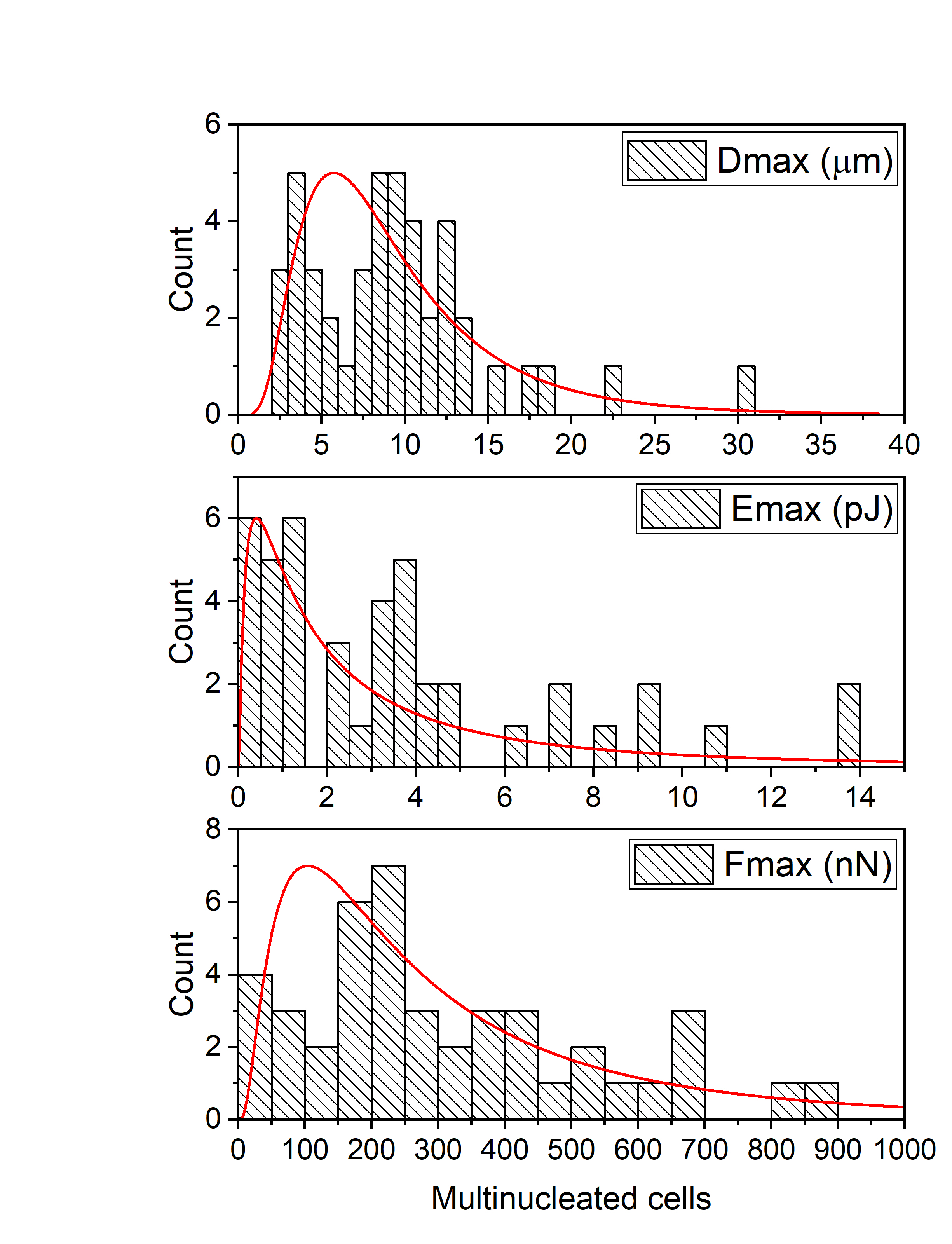


**Figure S6.** Histograms and lognormal fit of multi-nuclei cell characteristic SCFS parameter distributions.

**Table S3.** Lognormal fit parameters of *F*_max_, *E*_max,_ and *D*_max_ for the cell with multiple nuclei.

| -- | mu | sigma | mode | median | mean |
| --- | --- | --- | --- | --- | --- |
| Dmax | 2.09 | 0.58 | 5.73 | 8.07 | 9.57 |
| Emax | 0.8 | 1.3 | 0.41 | 2.23 | 5.21 |
| Fmax | 5.5 | 0.92 | 104.21 | 243.93 | 373.2 |


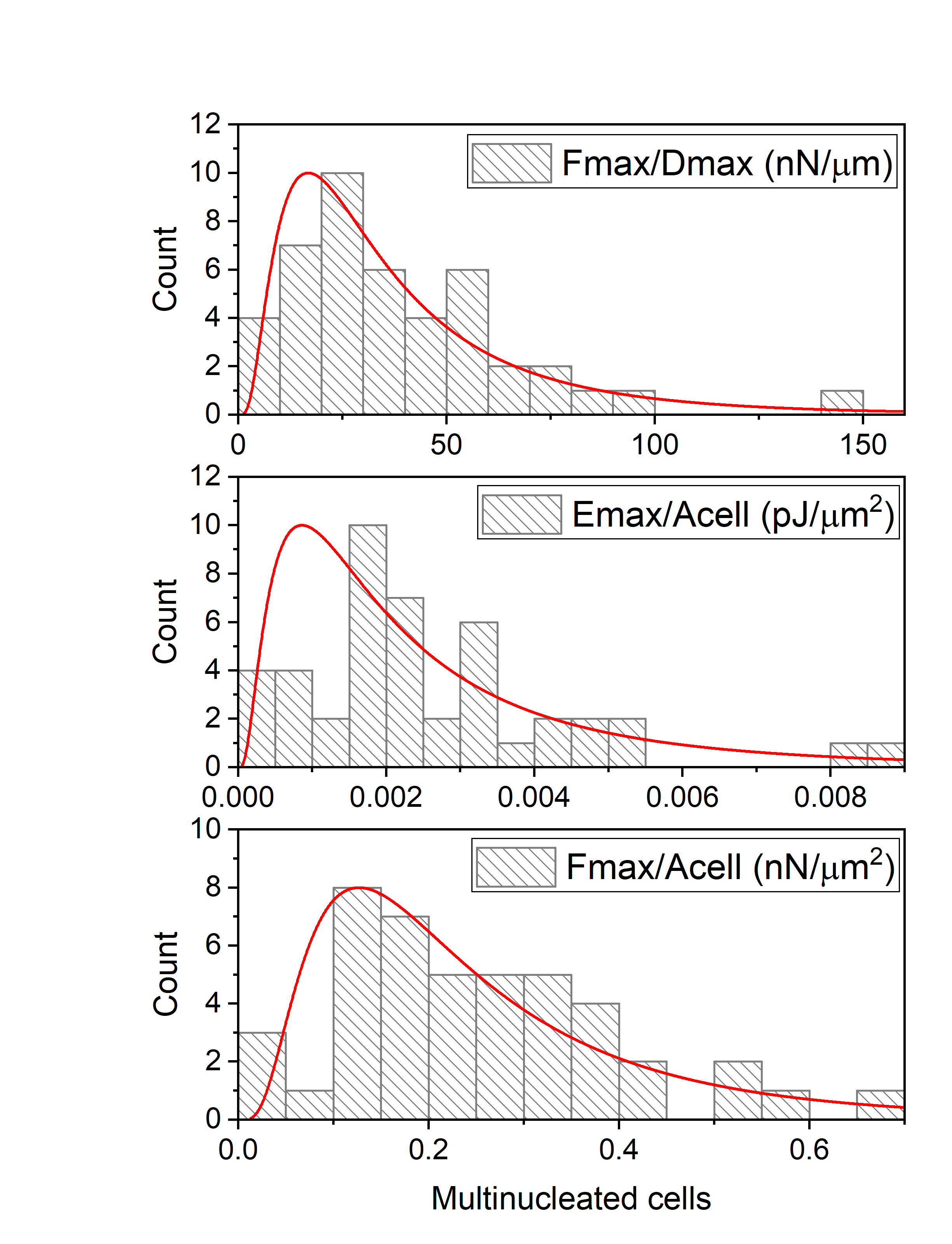


**Figure S7.** Histograms and lognormal fit of multi-nuclei cell area (*A*_cell_) normalized SCFS parameter and spring coefficient (*F*_max_/*D*_max_) distributions.

**Table S4.** Lognormal fit parameters of *F*_max_*/A*_cell_, *E*_max_*/A*_cell,_ and *F*_max_*/D*_max_ for cells with multiple nuclei.

| -- | mu | sigma | mode | median | mean |
| --- | --- | --- | --- | --- | --- |
| Fmax/Dmax | 3.41 | 0.77 | 16.81 | 30.24 | 40.56 |
| Emax/Acell | -6.27 | 0.9 | 9.00E-04 | 1.90E-03 | 0.003 |
| Fmax/Acell | -1.57 | 0.7 | 0.13 | 0.21 | 0.27 |
